# Increased risk of cardiovascular mortality by strict glycemic control (pre-procedural HbA1c < 6.5%) in Japanese medically-treated diabetic patients following percutaneous coronary intervention: a 10-year follow-up study

**DOI:** 10.1186/s12933-020-00996-8

**Published:** 2020-02-18

**Authors:** Takehiro Funamizu, Hiroshi Iwata, Yuya Nishida, Katsutoshi Miyosawa, Shinichiro Doi, Yuichi Chikata, Jun Shitara, Hirohisa Endo, Hideki Wada, Ryo Naito, Manabu Ogita, Tomotaka Dohi, Takatoshi Kasai, Shinya Okazaki, Kikuo Isoda, Katsumi Miyauchi, Hiroyuki Daida

**Affiliations:** 1 Department of Cardiovascular Medicine, Juntendo University Graduate School of Medicine, 2-1-1 Hongo, Bunkyo-ku, Tokyo, Japan; 2 Department of Metabolism and Endocrinology, Juntendo University Graduate School of Medicine, Tokyo, Japan; 3 Tokyo New Drug Research Laboratories, Kowa Company, Ltd., Tokyo, Japan; 4 Department of Cardiovascular Medicine, Juntendo University, Shizuoka Hospital, Shizuoka, Japan; 5 Department of Cardiovascular Medicine, Juntendo University Urayasu Hospital, Chiba, Japan

**Keywords:** Diabetes, HbA1c, Secondary prevention, Percutaneous coronary intervention, Cardiovascular death, Sudden death

## Abstract

**Background:**

In the secondary prevention of cardiovascular (CV) disease in patients with diabetes, an optimal level of HbA1c, the most widely-used glycemic control indicator, for favorable clinical consequences still remains to be established. This study assessed the association between preprocedural HbA1c level and CV mortality in Japanese diabetic patients undergoing percutaneous coronary intervention (PCI).

**Methods:**

This is a retrospective observational study using a single-center prospective PCI database involving consecutive 4542 patients who underwent PCI between 2000 and 2016. Patients with any antidiabetic medication including insulin at PCI were included in the analysis (n = 1328). We divided the patients into 5 and 2 groups according to HbA1c level; HbA1c: < 6.5% (n = 267), 6.5–7.0% (n = 268), 7.0–7.5% (n = 262), 7.5–8.5% (n = 287) and ≥ 8.5% (n = 244), and 7.0% > and ≤ 7.0%, respectively. The primary outcome was CV mortality including sudden death. The median follow-up duration was 6.2 years.

**Results:**

In the follow-up period, CV and sudden death occurred in 81 and 23 patients, respectively. While unadjusted Kaplan–Meier analysis showed no difference in cumulative CV mortality rate between patients binarized by preprocedural HbA1c 7.0%, analysis of the 5 groups of HbA1c showed significantly higher cumulative CV death in patients with HbA1c < 6.5% compared with those with 7.0–7.5% (P = 0.042). Multivariate Cox hazard analysis revealed a U-shaped relationship between preprocedural HbA1c level and risk of CV death, and the lowest risk was in the HbA1c 7.0–7.5% group (Hazard ratio of HbA1c < 6.5% compared to 7.0–7.5%: 2.97, 95% confidence interval: 1.33–7.25, P = 0.007). Similarly, univariate analysis revealed the lowest risk of sudden death was in the HbA1c 7.0–7.5% group.

**Conclusion:**

The findings indicate an increased risk of CV mortality by strict glycemic control (HbA1c < 6.5%) in the secondary prevention of CV disease in Japanese patients with medically-treated diabetes.

*Trial registration* This study reports the retrospective analysis of a prospective registry database of patients who underwent PCI at Juntendo University Hospital, Tokyo, Japan (Juntendo Physicians’ Alliance for Clinical Trials, J-PACT), which is publicly registered (University Medical Information Network Japan-Clinical Trials Registry UMIN-CTR 000035587).

## Background

Diabetes mellitus is a major socioeconomic burden worldwide [1] and it is the leading cause of atherosclerotic cardiovascular (CV) mortality and morbidity, including coronary, cerebral and peripheral artery disease, and heart failure [2]. To prevent macrovascular complications in diabetic patients, the multifactorial management of diabetes with other atherosclerotic risk factors such as smoking, obesity, hypertension, and lipid disorders is clinically important [3].

The level of glycated hemoglobin, hemoglobin A1c (HbA1c), reflects blood glucose levels over the preceding approximately 2 to 3 months [4]. As the measurement method of HbA1c has been clinically validated and internationally standardized [5], it has been widely accepted as an indicator of glycemic control, and accumulating evidence has recommended its routine monitoring in the care of diabetic patients [6]. Numerous previous studies have demonstrated that elevated HbA1c, generally more than 9.0%, is a strong risk factor for poor prognosis, such as CV mortality, not only in an entire cohort of type 2 diabetes patients [7], but also in diabetic patients with established coronary artery disease [8], heart failure [9], or chronic kidney disease (CKD) [10]. Accordingly, guidelines recommend glycemic control that has a target HbA1c level below 7.0% or 6.5% in the management of diabetes [6, 11]. Nevertheless, despite evidence indicating the merit of glycemic control to decrease the rates of microvascular complications [12, 13], three landmark trials showed no significant reduction in adverse CV events by intensive glycemic control in advanced diabetes with longer duration [14–16]. Moreover, patients with long-term diabetes, a known history of hypoglycemia, advanced atherosclerosis, or advanced age/frailty rather may benefit from a less aggressive HbA1c target [17]. Hypoglycemia has been shown to associate with an increased risk of all-cause mortality [18, 19], which casts doubt on very stringent glycemic control in diabetic patients. Moreover, although no substantial HbA1c lowering effect (< 0.5%) was observed in most of the recent trials of newer classes of antidiabetic agents, including dipeptidyl peptidase 4 (DPP-4) inhibitors [20–23], sodium glucose cotransporter 2 (SGLT2) inhibitors [24–26], and glucagon like peptide 1 (GLP-1) receptor agonists [27], the degrees of risk reduction of CV events were substantially different among these classes of anti-diabetic drugs. Accordingly, although most current primary and secondary prevention guidelines recommend HbA1c less than 6.5% or 7.0% to prevent poor CV outcomes in diabetic patients [6, 28], the optimal target level of HbA1c is still under intense debate [29]. Furthermore, evidence regarding a target level of HbA1c in the secondary prevention of CV events in patients with diabetes who have a history of macrovascular disease is still lacking and inconsistent. While the superiority of intensive glycemic control was not demonstrated in a subgroup of secondary prevention in the Action in Diabetes and Vascular Disease: Preterax and Diamicron Modified Release Controlled Evaluation (ADVANCE) trial [15], another study demonstrated more intensive lowering of HbA1c was beneficial to reduce the risk of CV events in patients with a history of macrovascular disease [30].

In this study, to explore and identify the optimal level of glycemic control in the secondary prevention of CV disease in patients with diabetes, we evaluated the association between the intensity or strictness of control represented by preprocedural HbA1c level at PCI and risk of subsequent CV mortality and sudden death in patients with any class of antidiabetic medication.

## Patients and Methods

This study was performed in accordance with the Declaration of Helsinki and with approval from the Institutional Review Board (IRB) of Juntendo University (IRB-ID: 17-170), and the prospective registry database of patients who underwent any PCI at Juntendo University Hospital, Tokyo, Japan (Juntendo Physicians’ Alliance for Clinical Trial, J-PACT) is publicly registered (University Medical Information Network Japan-Clinical Trials Registry, UMIN-CTR 000035587). Written informed consent was obtained from all participants for the J-PACT registry.

### Participants

This study is a retrospective analysis of a portion of the prospective single-center registry database of patients who underwent PCI at Juntendo University Hospital, Tokyo. The registry was launched in February 1984 (Juntendo Physicians’ Alliance for Clinical Trial, J-PACT). The registry database includes data regarding patient demographics, coronary artery lesions, PCI procedures, and devices used. Patients who underwent any type of percutaneous coronary artery intervention procedure, including thrombectomy, balloon angioplasty, and deployment of any type of coronary stent, were enrolled in the registry. In this study, the data of patients with a diagnosis of diabetes before PCI and prescribed any class of antidiabetic medication, such as insulin, GLP-1 receptor agonists and any oral antidiabetic drug, who underwent PCI for the first time between January 2000 and December 2016 was extracted from the registry database and validated by investigators.

In the 2000-2016 study period, consecutive 4542 patients who underwent PCI for the first time (index PCI) were registered in the database. After excluding patients with missing preprocedural HbA1c values (n = 94) and those undergoing chronic maintenance hemodialysis (n = 74), 1328 patients who were taking any anti-diabetic medication, including oral hypoglycemic agents and insulin, were enrolled in the study. In patients under chronic hemodialysis, the clinical usefulness of HbA1c as an indicator of glycemic control may need to be discussed separately, so we excluded that population [31]. To assess the prognostic impact of HbA1c level in this population, we divided the participants into 5 groups and 2 groups according to prespecified HbA1c values based on clinical relevance, specifically HbA1c < 6.5% (< 6.5%), 6.5% ≤ HbA1c < 7.0% (6.5–7%), 7.0% ≤ HbA1c < 7.5% (7–7.5%), 7.5% ≤ HbA1c < 8.5% (7.5–8.5%), and HbA1c ≥ 8.5% (≥ 8.5%), and HbA1c < 7.0% and ≥ 7.0%, respectively (Additional file 1: Figure S1).

### Follow-up

In this prospective PCI registry database, J-PACT, patient follow-up was based on chart review, as far as they were followed at Juntendo University Hospital. A prognosis survey questionnaire was mailed out every 5 years if they were transferred to other institutions. When there was no response to the questionnaire, further follow-up was conducted by phone. In cases in which no response was achieved by either, follow-up was terminated at the latest time point, at which their survival at our institution was confirmed, such as the last visit date to an outpatient clinic or the last day of any hospitalization. The median and range of the follow-up period since the index PCI were 6.2 and 0–10.0 years, respectively.

### Endpoints

The primary endpoint was CV mortality, which was defined as a composite of the following types of death; sudden death in which non-cardiac death could not be excluded, and death due to myocardial infarction, heart failure, cardiogenic shock, a cerebrovascular event, or aortic diseases. The secondary endpoint in this study was sudden death.

### Statistical analysis

Continuous variables are presented as the mean ± standard deviation or median with interquartile range (IQR) in accordance with the results of the Shapiro–Wilk normality test. Categorical variables are presented as the actual number and frequencies (%). Quantitative data across groups were compared using the ANOVA test or the Kruskal–Wallis test. Categorical variables were compared using the Fisher-exact test with the Chi squared test. Unadjusted Kaplan–Meier analysis evaluated the time to the cumulative cardiovascular mortality followed by the log-rank test for comparisons. The prognostic impact of preprocedural HbA1c level on CV mortality was assessed using univariate and multivariate Cox proportional hazards regression analyses. Multivariate analysis using the following two models calculated the hazard ratios (HR) with 95% confidence intervals (CI). In addition to the categorical analysis of HbA1c groups setting 7.0–7.5% as a reference control, Model 1 included the following variables; age, male gender, number of diseased vessels, systolic blood pressure, LDL-C, HDL-C, blood glucose and number of years with diabetes (covariates other than gender male were assessed as continuous variables, one standard deviation higher or 1 year longer), while Model 2 included age (a continuous variable), male gender, use of beta-blockers, ejection fraction (a continuous variable), hemoglobin, blood glucose, eGFR (a continuous variable), number of years with diabetes and insulin use. A P < 0.05 was considered to indicate statistical significance. Statistical analyses were performed using JMP version 11.2 (SAS Institute, Cary, NC).

## Results

### Baseline demographics and procedural characteristics among the 5 groups classified by preprocedural HbA1c level

The baseline and procedural characteristics of the 5 groups stratified according to HbA1c classification are summarized in Table 1. Patients with a lower HbA1c were older, more likely to have hypertension and/or CKD, but unlikely to present with acute coronary syndrome. Patients in the groups with a lower HbA1c level had a lower body mass index (BMI) and lower levels of serum lipid parameters, including triglycerides, high density lipoprotein cholesterol (HDL-C) and low density lipoprotein cholesterol (LDL-C), and high-sensitivity C-reactive protein (hs-CRP). Insulin was less frequently used in patients with lower HbA1c levels. The proportions of patients with multivessel disease, complex lesions, and stent implantation were similar among the groups. Nutritional status represented by serum albumin as well as geriatric nutritional risk index (GNRI) was not different among the groups. Similarly, there was no significant difference in the proportion of administration of drugs other than antidiabetic medications, statins, beta-blockers, angiotensin converting enzyme (ACE) inhibitors, and angiotensin receptor blockers (ARB).

**Table 1:** Baseline clinical characteristics of the study population

	Overall	HbA1c < 6.5%	6.5 ≤ HbA1c < 7.0%	7.0 ≤ HbA1c < 7.5%	7.5 ≤ HbA1c < 8.5%	HbA1c ≥ 8.5%	P-value
	(n = 1328)	(< 6.5%)	(6.5–7%)	(7–7.5%)	(7.5–8.5%)	(≥ 8.5%)	
		(n = 267)	(n = 268)	(n = 262)	(n = 287)	(n = 244)	
Age, years-old	66.7 ± 9.7	68.3 ± 9.8	68.5 ± 8.4	67.5 ± 9.1	66.2 ± 9.6	62.6 ± 10.8	*< 0.001*
BMI, kg/m^2^	24.5 ± 3.6	24.1 ± 3.2	24.7 ± 3.8	24.2 ± 3.5	24.8 ± 3.6	24.9 ± 3.7	*0.018*
Male, n (%)	1079 (81.3)	222 (83.2)	210 (78.4)	217 (82.8)	239 (83.3)	191 (78.3)	0.32
Hypertension, n (%)	994 (74.9)	215 (80.5)	208 (77.6)	197 (75.2)	206 (71.8)	168 (68.9)	*0.019*
Dyslipidemia, n (%)	1246 (93.8)	253 (94.8)	248 (92.5)	247 (94.3)	272 (94.8)	226 (92.6)	0.68
Current or ex-smoker, n (%)	348 (26.3)	48 (18.0)	62 (23.2)	63 (24.1)	80 (28.0)	95 (38.9)	*< 0.001*
Family history, n (%)	365 (27.6)	73 (27.4)	75 (28.0)	73 (28.0)	67 (23.5)	77 (31.6)	0.36
ACS presentation, n (%)	344 (25.9)	60 (22.5)	52 (19.4)	61 (23.3)	82 (28.6)	89 (36.5)	*< 0.001*
Number of diseased vessels	2.0 ± 0.8	2.1 ± 0.8	2.0 ± 0.8	2.0 ± 0.9	2.0 ± 0.8	1.9 ± 0.8	0.60
Complex lesion, n (%)^a^	1095 (88.3)	224 (89.2)	225 (88.9)	222 (89.5)	226 (85.0)	198 (89.2)	0.45
Use of Stent, n (%)	1202 (90.5)	251 (94.0)	238 (88.8)	232 (88.6)	261 (90.9)	220 (90.2)	0.20
SBP, mmHg	135.9 ± 23.4	136.3 ± 22.8	137.2 ± 23.6	136.1 ± 24.3	135.7 ± 22.1	134.0 ± 24.3	0.62
DBP, mmHg	73.0 ± 14.0	73.5 ± 13.5	73.0 ± 13.5	72.6 ± 15.2	72.5 ± 13.7	73.5 ± 13.8	0.86
Ejection fraction, (%)	60.9 ± 12.8	61.4 ± 11.3	60.8 ± 12.8	61.6 ± 13.6	60.8 ± 13.6	59.7 ± 12.7	0.59
Hemoglobin, g/dL	13.3 ± 1.8	13.1 ± 1.9	13.1 ± 1.8	13.3 ± 1.6	13.5 ± 1.7	13.6 ± 2.0	*< 0.001*
HbA1c, %	7.5 ± 1.3	6.1 ± 0.3	6.8 ± 0.1	7.3 ± 0.2	7.9 ± 0.3	9.7 ± 1.2	*< 0.001*
TC, mg/dL	176.3 ± 39.2	168.0 ± 37.8	172.9 ± 35.2	172.2 ± 37.5	178.1 ± 38.8	191.6 ± 43.0	*< 0.001*
LDL-C, mg/dL	105.8 ± 33.2	100.5 ± 35.8	103.0 ± 28.6	102.6 ± 31.2	105.6 ± 31.8	118.4 ± 35.6	*< 0.001*
HDL-C, mg/dL	43.2 ± 13.1	42.3 ± 12.1	43.5 ± 12.5	44.2 ± 12.0	42.9 ± 13.1	42.9 ± 15.7	0.55
TG, mg/dL	118 (87.0–165)	113 (83.0–150)	116 (87.3–162)	113 (82.0–152)	122 (90.0–181)	129 (93.0–185)	*0.0024*
Blood glucose, mg/dL	146.0 ± 64.7	117.7 ± 36.7	126.4 ± 41.7	140.4 ± 53.3	153.0 ± 63.4	196.3 ± 87.9	*< 0.001*
eGFR, mL/min/1.73 m^2^	72.5 ± 22.9	70.0 ± 20.1	70.0 ± 23.4	72.1 ± 23.7	73.7 ± 21.8	77.2 ± 25.1	*0.0014*
CKD (stage 3–5), n (%)	369 (27.8)	83 (31.1)	84 (31.3)	75 (28.6)	70 (24.4)	57 (23.4)	0.12
hs-CRP, mg/dL	0.11 [0.04–0.32]	0.10 [0.03–0.25]	0.10 [0.04–0.28]	0.09 [0.04–0.22]	0.13 [0.05–0.37]	0.20 [0.06–0.60]	*< 0.001*
Alb, g/dL	3.91 ± 0.46	3.92 ± 0.42	3.90 ± 0.47	3.92 ± 0.46	3.92 ± 0.46	3.88 ± 0.52	0.86
GNRI	103.1 [96.0–109.7]	102.3 [95.9–108.0]	103.9 [96.0–110.3]	101.8 [94.2–109.5]	103.9 [97.0–110.7]	104.1 [96.4–110.2]	0.18
Diabetes duration, years	12 [5.0–20]	10 [3.0–20]	13 [5.0–20]	12 [5.0–20]	12 [6.0–20]	12 [5.0–20]	0.18
Glucose-lowering drugs							
Sulfonylurea, n (%)	564 (42.5)	99 (37.1)	106 (39.6)	101 (38.6)	141 (49.1)	117 (48.0)	*0.0074*
Metformin, n (%)	258 (19.4)	41 (15.4)	48 (17.9)	61 (23.3)	61 (21.3)	47 (19.3)	0.18
Thiazolidinedione, n (%)	169 (12.7)	32 (12.0)	40 (14.9)	34 (13.0)	41 (14.3)	22 (9.0)	0.29
DPP4 inhibitor, n (%)	274 (20.6)	76 (28.5)	70 (26.1)	55 (21.0)	55 (19.2)	18 (7.4)	*< 0.001*
α-Glucosidase inhibitor, n (%)	516 (38.9)	113 (42.3)	104 (38.8)	97 (37.0)	112 (39.0)	90 (36.9)	0.71
Glinides, n (%)	144 (10.8)	30 (11.2)	31 (11.6)	36 (13.7)	26 (9.1)	21 (8.6)	0.32
Insulin, n (%)	427 (32.2)	37 (13.9)	69 (25.8)	81 (30.9)	99 (34.5)	141 (57.8)	*< 0.001*
Other drugs							
Statin, n (%)	1006 (75.8)	203 (76.0)	215 (80.2)	192 (73.3)	220 (76.7)	176 (72.1)	0.23
Beta-blocker, n (%)	631 (48.1)	132 (49.8)	133 (49.8)	113 (43.6)	135 (48.0)	118 (49.4)	0.58
ACEI/ARB, n (%)	724 (55.2)	149 (56.2)	151 (56.6)	139 (53.7)	145 (51.6)	140 (58.6)	0.54

*BMI* body mass index, *ACS* acute coronary syndrome, *SBP* systolic blood pressure, *DBP* diastolic blood pressure, *TC* total cholesterol, *LDL-C* low density lipoprotein-cholesterol, *HDL-C* high density lipoprotein-cholesterol, *TG* triglycerides, *FBG* fasting blood glucose, *eGFR* estimated glomerular filtration rate, *CKD* chronic kidney disease, *hs-CRP* high-sensitivity C-reactive protein, *Alb* albumin, *GNRI* geriatric nutritional risk index, *DPP4* dipeptidyl peptidase 4, *ACEI* angiotensin-converting enzyme inhibitor, *ARB* angiotensin receptor blocker

^a^Complex lesion defined as ACC/AHA type B2 or type C lesion. *ACC/AHA* American College of Cardiology/American Heart Association

### Cardiovascular mortality rate and HbA1c level

During follow-up periods up to 10 years since the first PCI, 216 all-cause deaths out of 1328 patients (16.3%) and 81 CV deaths (6.1%) were identified. The causes of the CV deaths included sudden death (n = 23, 28.4% in CV death), death due to acute myocardial infarction (n = 8, 9.9%), heart failure and cardiogenic shock (n = 26, 32.1%), cerebrovascular event (n = 16, 19.8%), and other cardiovascular causes, such as aortic diseases (n = 8, 9.9%). Among the 5 groups, the crude incidences of CV and sudden death were the lowest in the HbA1c 7.0–7.5% group, although no statistically significant difference was revealed by the Fisher exact test followed by the Chi squared test (Table 2).

**Table 2:** Overall incidence of cardiovascular events (per 1000 person-years)

	Overall	HbA1c < 6.5%	6.5 ≤ HbA1c < 7.0%	7.0 ≤ HbA1c < 7.5%	7.5 ≤ HbA1c < 8.5%	HbA1c ≥ 8.5%	P-value
	(n = 1328)	(< 6.5%)	(6.5–7%)	(7–7.5%)	(7.5–8.5%)	(≥ 8.5%)	
		(n = 267)	(n = 268)	(n = 262)	(n = 287)	(n = 244)	
All-cause death, n (/1000 person-years)	216 (27.2)	48 (33.3)	45 (30.9)	38 (24.2)	44 (25.0)	41 (23.8)	0.83
Cardiovascular death, n (/1000 person-years)	81 (10.2)	21 (14.6)	12 (8.2)	11 (7.0)	17 (9.6)	20 (11.6)	0.18
Sudden death, n (/1000 person-years)	23 (2.9)	8 (5.6)	2 (1.4)	2 (1.3)	4 (2.3)	7 (4.1)	0.11
Acute myocardial infarction, n (/1000 person-years)	8 (1.0)	2 (1.4)	1 (0.7)	0 (0)	3 (1.7)	2 (1.2)	0.55
Heart failure and cardiogenic shock, n (/1000 person-years)	26 (3.3)	3 (2.1)	5 (3.4)	5 (3.2)	5 (2.8)	8 (4.6)	0.51
Cerebrovascular event, n (/1000 person-years)	16 (2.0)	6 (4.2)	4 (2.7)	1 (0.6)	4 (2.3)	1 (0.6)	0.24
Other cardiovascular causes, n (/1000 person-years)	8 (1.0)	2 (1.4)	0 (0)	3 (1.9)	1 (0.6)	2 (1.2)	0.48

### Cumulative cardiovascular mortality rate stratified by HbA1c level

**Fig. 1:**
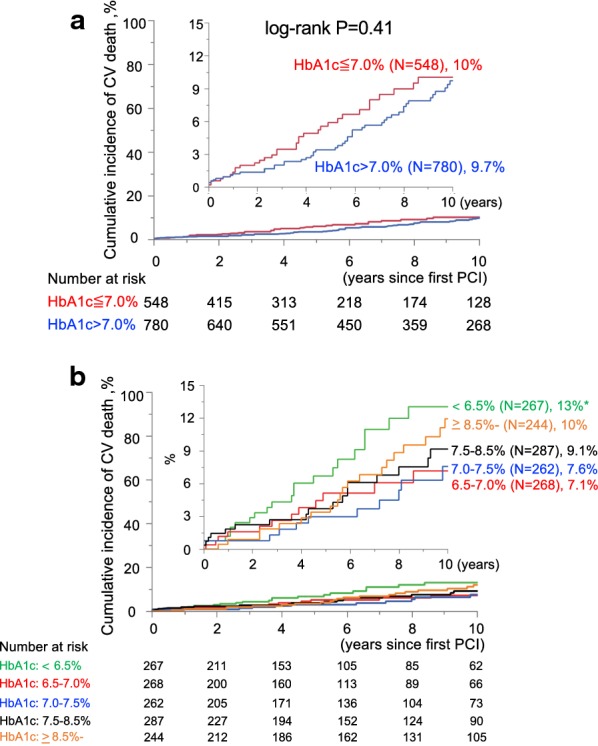
Cumulative cardiovascular mortality rates among groups stratified by preprocedural HbA1c. **a** Kaplan–Meier curves of 2 diabetic patient groups with or without preprocedural HbA1c below 7.0%. No significant difference in the cumulative incidence of cardiovascular death in diabetic patients binarized by HbA1c 7.0%; HbA1c > 7.0% group vs. HbA1c ≤ 7.0% group (Log-rank comparison, P = 0.41). Percent indicates the cumulative incidence of CV death at 10 years of follow-up in each group. **b** Kaplan–Meier curves of 5 groups stratified by preprocedural levels of HbA1c. Participants were divided into 5 groups according to their HbA1c values; < 6.5% (n = 267), 6.5–7.0% (n = 268), 7.0–7.5% (n = 262), 7.5–8.5% (n = 287), and ≥ 8.5% (n = 244). The lowest cumulative incidence of cardiovascular death was in the HbA1c 7.0–7.5% group, which was significantly lower than that in the HbA1c < 6.5% group. Percent indicates the cumulative incidence of CV death at 10 years of follow-up in each group. * indicates P < 0.05 by log-rank comparison vs. 7.0% ≤ HbA1c < 7.5%. *CV death* cardiovascular death, *HbA1c* glycated hemoglobin, *PCI* percutaneous coronary intervention

Unadjusted Kaplan–Meier analysis followed by the log-rank comparison test showed no significant difference in the cumulative CV mortality rate when the study participants were binarized by HbA1c 7.0% into a group with HbA1c > 7.0% vs. HbA1c ≤ 7.0% (P = 0.41), although the Kaplan–Meier curve of the group with HbA1c ≤ 7.0% was consistently higher than that of HbA1c > 7.0% (Figure 1a). Kaplan–Meier curves of cumulative CV mortality of the 5 patient groups stratified by preprocedural HbA1c levels (< 6.5%, 6.5–7.0%, 7.0–7.5%, 7.5–8.5% and ≥ 8.5%) showed that the lowest cumulative CV mortality rate was in the HbA1c 7.0–7.5% group, which was significantly lower than that of HbA1c < 6.5% (P = 0.042 by log-rank comparison) (Fig. 1b). Kaplan–Meier analyses in the HbA1c < 6.5% group showed that the cumulative CV mortality rate was significantly higher in patients with low body weight, insulin use, and low albumin compared to those without (Additional file 1: Figure S2), indicating that they are potential risk factors for cardiovascular death in medically-treated diabetic patients with HbA1c < 6.5%.

### Adjusted prognostic impact of preprocedural HbA1c level for cardiovascular and sudden death

**Fig. 2:**
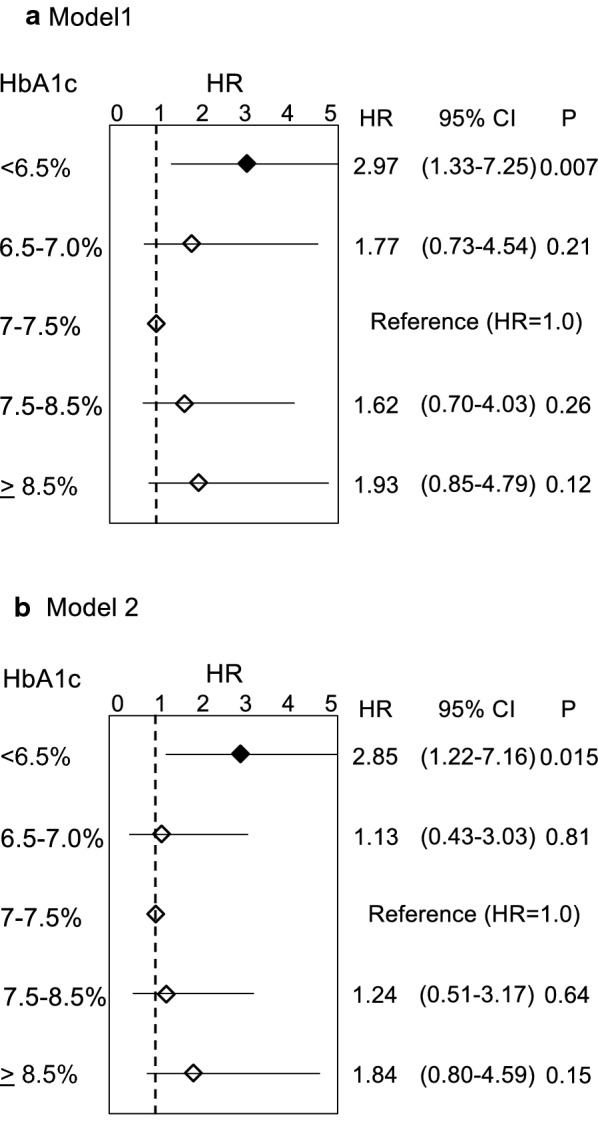
Adjusted hazard ratios for incidence of cardiovascular death by HbA1c categories in different models. **a** Model 1: Hazard ratios by categorized HbA1c adjusted by age (1 year older), gender male, number of diseased vessels, systolic blood pressure (1SD higher), LDL-cholesterol (1SD higher), HDL-cholesterol (1SD higher), blood glucose (1SD higher) and diabetes duration (1 year longer). U-shaped risk for cardiovascular death by categorized HbA1c level; the risk was lowest in the group with HbA1c 7.0–7.5%. Compared with that group, the risk of HbA1c < 6.5% was significantly higher. **b** Model 2: Hazard ratios by categorized HbA1c adjusted by age (1 year older), gender male, use of beta-blockers, ejection fraction (1SD higher), serum hemoglobin (1SD higher), blood glucose (1SD higher), eGFR (1SD higher), diabetes duration (1 year longer) and insulin use. Consistent with Model 1, a U-shaped relationship between preprocedural HbA1c and risk of cardiovascular mortality is shown. The lowest hazard ratio was observed in the group with HbA1c 7.0–7.5%. *HbA1c* glycated hemoglobin (%), *SD* standard deviation, *eGFR* estimated glomerular filtration rate

To address the prognostic impact of the preprocedural HbA1c level in diabetic patients following PCI independently, we performed categorical univariate and multivariate Cox proportional hazard analyses of preprocedural HbA1c 7.0–7.5% group as a control reference using two models for predicting CV death. Covariates included in multivariate analysis were selected by combining the clinical and biological plausibility with the results of univariate analyses (Additional file 1: Table S1). In addition to the categorical analysis of HbA1c level, Model 1 included the following variables; age, male gender, number of diseased vessels, systolic blood pressure, LDL-C, HDL-C, blood glucose and number of years with diabetes (covariates other than gender male were assessed as continuous variables, one standard deviation higher or 1 year longer), while Model 2 included age (a continuous variable), male gender, use of beta-blockers, ejection fraction (a continuous variable), hemoglobin, blood glucose, eGFR (a continuous variable), number of years with diabetes and insulin use. Multivariate analyses using these two models continuously showed that the hazard ratios for CV death were the lowest in patients with HbA1c 7.0–7.5%, and were higher in patients with the lowest (< 6.5%) and highest (≥ 8.5%) categories of HbA1c, indicating the relationship between the adjusted risk for CV death and preprocedural HbA1c was not linear, but rather U-shaped (Fig. 2a, b) (Additional file 1: Table S2). Moreover, as a continuous variable, one standard deviation (1SD) higher HbA1c was not associated with the risk of CV mortality by univariate and multivariate Cox regression analysis, while 1SD higher in blood glucose, hemoglobin, diabetes duration, eGFR, and ejection fraction were significantly associated with increased and reduced risk of subsequent cardiovascular mortality (Additional file 1: Table S3), indicating a non-linear relationship between the preprocedural HbA1c level and the risk for CV death. For further clarification of the prognostic impact of preprocedural HbA1c, a categorical univariate Cox proportional hazard analysis was used to calculate the hazard ratios for sudden death specifically. Adjusted multivariate analysis was not performed because the number of sudden deaths within the observational period (n = 23) in this study was limited. Consequently, similar to multivariate adjusted Cox proportional hazard analysis for cardiovascular mortality, the relationship between preprocedural HbA1c and the risk of sudden death was U-shaped (Additional file 1: Figure S3). Moreover, there were no significant relationships between categorized HbA1c with any of all-cause, cancer associated, and non-CV mortalities (Additional file 1: Figure S4).

## Discussion

This single-center observational analysis involving 1328 diabetic patients who underwent PCI demonstrated that the association between preprocedural HbA1c level before PCI and the CV mortality rate was U-shaped. In an effort to more clearly elucidate the prognostic impact of HbA1c level in this population, this study included patients who had been diagnosed with diabetes before PCI and had been using any antidiabetic medication. While HbA1c was not associated with the risk of CV death as a continuous variable, unadjusted Kaplan–Meier analysis, as well as adjusted Cox proportional hazard analysis, showed the lowest CV mortality risk was in the HbA1c 7.0–7.5% subgroup. A similar U-shaped correlation between preprocedural HbA1c level and the risk of sudden death was also observed.

As an indicator of averaged glycemic control in the prior 2–3 months, HbA1c testing has been applied to a broad range of clinical settings of diabetes care since its screening, diagnosis, follow-up, assessment of treatment effect, and risk stratification. Accordingly, the latest guidelines consistently recommend routine testing of HbA1c in all patients with diabetes [6, 28]. It has been well established that poor glycemic control is harmful in diabetic patients who are complicated by various types of coronary artery disease [8], such as impaired microvascular circulation [32].

However, the evidence for HbA1c-guided strict diabetic control for preventing macrovascular complications is limited, while an association between higher HbA1c and increased risk of microvascular complications has been previously demonstrated [33, 34]. Even though strict and multifactorial comprehensive management of atherosclerotic risk factors, such as obesity, smoking, lipid disorders and hypertension in secondary prevention of CV events in patients with diabetes is recommended [3], the prognostic implication of HbA1c level is still controversial and only a limited number of studies have addressed it with respect to CV mortality in patients with established coronary artery disease who underwent PCI. Moreover, most of previous studies binarized using 7.0% HbA1c and failed to show any significant differences in the incidences of adverse CV events between the groups [35–40], a finding which is consistent with that of this study. For a detailed evaluation of the prognostic impact of preprocedural HbA1c level, this study divided the participants into 5 groups with an almost equal distribution of patient numbers in each group, < 6.5%, 6.5–7.0%, 7.0–7.5%, 7.5–8.5% and ≥ 8.5%, allowing us to focus on the risk of lower HbA1c levels. Although two studies of large-scale registries that included diabetic patients with both primary and secondary prevention of CV disease have described a U-shaped relationship between HbA1c level and the risk of all-cause mortality [41, 42], a single center observational study in patients following PCI showed a linear correlation in a group of non-insulin users [8]. Similar to the present study, that study divided participants into 5 groups according to preprocedural HbA1c level [8]. However, the HbA1c thresholds in the two studies were substantially different. They put more focus on the risk of relatively higher HbA1c levels and showed a linear increased risk of all-cause death with elevating procedural HbA1c level in non-insulin users. However, in their study, since a large portion of the participants (44%) were assigned to the HbA1c < 7.0% group without further classification, it may mask the risk of low HbA1c and lead to substantially divergent conclusions.

Two previous landmark trials, the ACCORD and ADVANCE trials [14, 15], failed to show the superiority of HbA1c-guided intensive glycemic control targeting HbA1c below 6.0% or 6.5% compared to standard control, with respect to the occurrence of all-cause death, as well as the composite of CV mortality, myocardial infarction, and stroke, and the higher incidence of hypoglycemia in the group with intensive glycemic control group was assumed to be responsible for the results in these studies. Similarly, extended follow-up (10 and 15 years) in the Veterans Affairs Diabetes Trial (VADT) showed that intensive glucose control had no significant cardiovascular-related mortality effect compared to standard control, while a lower incidence of the composite of cardiovascular events, myocardial infarction, stroke, heart failure and amputation for ischemic gangrene and CV mortality was observed in the intensive glycemic control group [43, 44]. Additionally, the benefit of intensive glycemic control in critical patients admitted to the intensive care unit (ICU) is still a topic of debate [45, 46]. Moreover, a legacy effect of early glycemic control on the long-term prognosis of diabetic patients has been suggested [47], although it is still controversial [44]. Moreover, the associations between a higher incidence of hypoglycemia in diabetic patients and CV mortality [48] and critical arrhythmia [49] were previously described, which might be a possible explanation for the findings regarding sudden and CV death in patients with a lower HbA1c below 6.5% in this study. Sympathoadrenal activation by hypoglycemia has long been known to induce a prolonged QT interval and cardiac repolarization [50, 51], resulting in critical ventricular arrhythmia and sudden cardiac death [52].

Caution may be needed in the interpretation of the findings in this study that the hemoglobin value, an established prognostic indicator in diabetic patients [53] and those who subsequently undergo PCI [54], may cause potential bias in the prognostic implication of HbA1c. Conditions that affect red blood cell turnover, such as hemolytic and other types of anemia, may result in discrepancies between the HbA1c level and true glycemic control. However, the translation of other measures of average glycemia independent from hemoglobin value, such as fructosamine and 1,5-anhydroglucitol, into prognostic significance in this population are more unclear than HbA1c. In this study, the prognostic implication of low HbA1c was assessed by multivariate analysis with adjustment of covariates that included hemoglobin value, and the difference between the groups with HbA1c below 6.5% and the 7.0–7.5% group was still significant.

The updated recommendations by the American Diabetes Association (ADA) and European Association for the Study of Diabetes (EASD) of glycemic control suggests an approach consisting of the individualization of glycemic targets that depend on the individual patient’s age, background factors, and vascular complications. In particular, less stringent targets with relatively higher HbA1c levels were recommended for patients with advanced macrovascular complications, and our findings of the lowest rates of CV mortality and sudden death in patients with HbA1c 7.0–7.5% may satisfy such recommendations in these guidelines [6, 55].

This study has several limitations to consider. First, since it was retrospectively analyzed a single center prospective registry database involving a relatively smaller number of Japanese participants without any randomization, unaccounted confounding factors, which were not recorded or were not included in the model, may mediate and lead to the outcomes, although we have adjusted for known confounding factors. Moreover, the number of sudden deaths was limited (n = 23) in this study and thus insufficient for multivariate analysis. Although the univariate analysis findings were significant, the association of HbA1c and sudden death may need to be evaluated in a larger scale study. Second, HbA1c level was measured at a single time point, before the procedure in this study. Therefore, the extent of glycemic control after PCI is unknown. Although we considered that glycemic control after PCI was consistent with that before the procedure, the risk of hypoglycemia and poor prognosis may need to be evaluated by a study with multiple sampling time points of HbA1c. Furthermore, recent studies have shown variability in the HbA1c or blood glucose level have a significant impact on the risk of mortality [56, 57] and macrovascular diabetic complications [58]. Therefore, given that its levels at multiple time points were available in this study, the prognostic effect of temporal changes in HbA1c level that reflect long-term glycemic control and its variability may be able to clarify the prognostic implication, as well as the therapeutic goal in the treatment of diabetes following PCI for preventing adverse events. Third, since individuals who underwent treatment after January 2017 were not included in the analysis in this study due to insufficient follow-up duration, very few patients had received new generation antidiabetic drugs, such as GLP-1 receptor agonists (n = 4, 0.3%) and SGLT2 inhibitors (n = 7, 0.5%), which have recently been shown to substantially lower the risks of cardiovascular mortality and morbidity. Although large-scale randomized trials have indicated the prognostic merits of these drugs were mostly independent of HbA1c reduction [24, 27, 59], the prognostic impact of preprocedural HbA1c in this study might be overwritten by these two classes of antidiabetic drugs, in consideration of their significant prognostic merit. Thus, future studies may be needed to evaluate the prognostic implication of HbA1c levels in patients receiving these drugs. Lastly, the mechanistic insight of the association between low HbA1c levels below 6.5% and higher CV mortality needs to be further elucidated, although we addressed the potential correlation between low HbA1c and increased risk of sudden death. Even with these limitations, the strengths of the present study include a detailed evaluation of the cause of death, such as CV death and sudden death. Therefore, the present findings may have clinical implications in the glycemic targets of diabetic patients undergoing PCI.

## Conclusions

This study has demonstrated that both low and high preprocedural HbA1c levels in Asian diabetic patients who underwent PCI are associated with an increased risk of subsequent long-term CV mortality, sudden death in particular, and the HbA1c control range with lowest risk was 7.0–7.5%. The findings in this study may suggest there are limitations to the HbA1c-guided intensive control of diabetes for the purpose of reducing risk of macrovascular events in the secondary prevention of CV disease in diabetic patients.

## Supplementary information

**Additional file 1.** Additional figures and tables.

## Data Availability

The datasets used and/or analyzed during the current study are available from the corresponding author on reasonable request.

## Abbreviations

HbA1c: Glycated hemoglobin
CV: Cardiovascular
PCI: Percutaneous coronary intervention
CKD: Chronic kidney disease
DPP-4: Dipeptidyl peptidase 4
SGLT2: Sodium glucose cotransporter 2
GLP-1: Glucagon like peptide 1
IRB: Institutional review board
IQR: Interquartile range
HR: Hazard ratios
CI: Confidence intervals
1SD: One standard deviation
eGFR: Estimated glomerular filtration rate
BMI: Body mass index
HDL-C: High density lipoprotein cholesterol
LDL-C: Low density lipoprotein cholesterol
hs-CRP: High-sensitivity C-reactive protein
ACE: Angiotensin converting enzyme
ARB: Angiotensin receptor blockers
