# Supplementary material for: Increased risk of cardiovascular mortality by strict glycemic control (pre-procedural HbA1c < 6.5%) in Japanese medically-treated diabetic patients following percutaneous coronary intervention: a 10-year follow-up study
Source: Cardiovasc Diabetol. 2020 Feb 18;19:21. doi: 10.1186/s12933-020-00996-8 (PMC7027034; doi:10.1186/s12933-020-00996-8)
Supplement: Supplementary file 1 — Additional file 1. Additional figures and tables. [file 12933_2020_996_MOESM1_ESM.docx]

**Additional file**

**Additional Figure S1**

**Additional Figure S2**

**
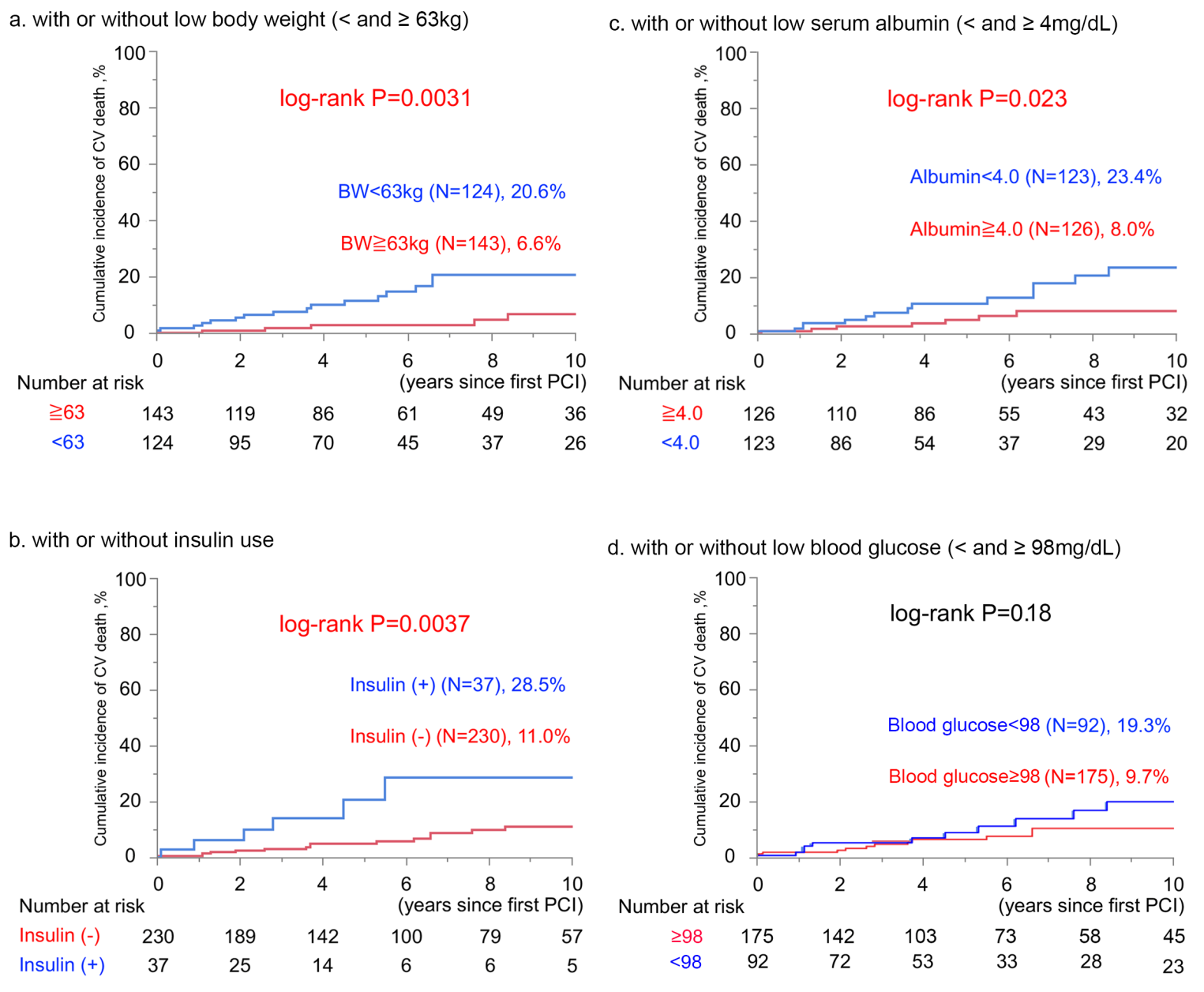
**

**Additional Figure S3**

**Additional Figure S4**

**A. All-cause mortality**

**B**. **Cancer associated mortality**

**C. Non-CV mortality**

**Additional Figure legends**

**Additional Figure S1: Study flowchart of the patient enrollment**

This study enrolled 1,328 diabetic patients who underwent PCI for the first time at Juntendo University Hospital between January 2000 and December 2016 and were administered any antidiabetic medication. After exclusion of patients with no HbA1c data, any diabetic medication, or undergoing chronic hemodialysis, 1328 patients were included in the analysis. PCI: percutaneous coronary intervention; HbA1c: glycated hemoglobin.

**Additional Figure S2: Subgroup Kaplan-Meier analyses in patients with HbA1c <6.5%**

Kaplan-Meier analysis in patients with HbA1c<6.5% stratified by body weight (BW) (a), insulin use (b), serum albumin (c) and blood glucose (d). In this subpopulation, the cumulative cardiovascular mortality rates in patients with low BW (< median, 63 kg), insulin use and low serum albumin (< median, 4.0 mg/dL) were higher, while there was no difference between patients with and without low blood glucose (< lowest tertile, 98 mg/dL).

**Additional Figure S3: Unadjusted hazard ratios of categorized HbA1c for sudden death**

Similar to the relationship between categorized HbA1c and cumulative cardiovascular mortality rate, univariate Cox proportional hazard analysis showed a U-shaped relationship between preprocedural HbA1c and sudden death. HbA1c: glycated hemoglobin; HR: hazard ratio; CI: confidence interval.

**Additional Figure S4:** **Multivariate Cox proportional hazard analysis for all-cause, cancer associated, and non-CV mortality of categorized HbA1c**

No significant relationships between categorized HbA1c with all-cause, cancer associated, and non-CV mortalities were observed, although there was a slight U-shaped-like relationship in all-cause mortality.

**Additional Table S1: Univariate analysis for cardiovascular mortality**

|  | HR^1^ | 95% CI^2^ | P-value |
| --- | --- | --- | --- |
| **Age (1 year older)** | **1.06** | **1.03-1.08** | **<0.001** |
| Male gender | 0.76 | 0.47-1.31 | 0.32 |
| Smoking history | 0.92 | 0.58-1.47 | 0.71 |
| Dyslipidemia | 0.71 | 0.36-1.59 | 0.37 |
| **Beta-blockers** | **1.60** | **1.02-2.55** | **0.038** |
| ACEIs/ARBs^3^ | 1.04 | 0.67-1.63 | 0.87 |
| **Number of diseased vessels (1 more vessel)** | **1.40** | **1.07-1.85** | **0.014** |
| Systolic blood pressure (1SD^4^, 23.4 mmHg higher) | 0.92 | 0.72-1.16 | 0.48 |
| **Ejection fraction (1SD, 12.8% higher)** | **0.57** | **0.47-0.70** | **<0.001** |
| **Serum hemoglobin (1SD, 1.8 g/dl higher)** | **0.51** | **0.41-0.64** | **<0.001** |
| Serum LDL-C^5^ (1SD, 33.2 mg/dl higher) | 1.04 | 0.83-1.28 | 0.73 |
| Serum HDL-C^6^ (1SD, 13.1 mg/dl higher) | 0.93 | 0.73-1.15 | 0.53 |
| **Serum triglycerides (1SD, 78.1 mg/dl higher)** | **0.76** | **0.56-0.99** | **0.038** |
| **Blood glucose (1SD, 64.7 mg/dl higher)** | **1.27** | **1.05-1.50** | **0.016** |
| **eGFR^7^ (1SD, 22.9 ml/min/1.73m^2^ higher)** | **0.49** | **0.38-0.62** | **<0.001** |
| **Diabetes duration (1 year longer)** | **1.05** | **1.03-1.07** | **<0.001** |
| **Insulin** | **1.98** | **1.27-3.06** | **0.0026** |
| Sulfonylurea | 0.91 | 0.59-1.42 | 0.69 |
| Metformin | 1.05 | 0.58-1.78 | 0.87 |
| α-glucosidase inhibitors | 1.00 | 0.64-1.55 | 0.99 |

1: HR: hazard ratio, 2: CI: confidence interval, 3: ACEIs/ARBs: angiotensin converting enzyme inhibitors/ angiotensin receptor blockers, 4: SD: standard deviation, 5: LDL-C: low density lipoprotein-cholesterol, 6: HDL-C: high density lipoprotein-cholesterol, 7: eGFR: estimated glomerular filtration rate

**Additional Table S2:** **Adjusted multivariate Cox proportional hazard analysis using 2 models**

**Model 1**

|  | HR^1^ | 95%CI^2^ | P-value |
| --- | --- | --- | --- |
| **Age (1 year older)** | **1.04** | **1.01-1.07** | **0.017** |
| Male gender | 0.81 | 0.47-1.45 | 0.47 |
| Number of diseased vessels (1 more vessel disease) | 1.22 | 0.92-1.64 | 0.17 |
| SBP^3^ (1SD^4^ higher) | 0.97 | 0.76-1.22 | 0.80 |
| LDL-C^5^ (1SD higher) | 1.04 | 0.82-1.31 | 0.73 |
| HDL-C^6^ (1SD higher) | 0.94 | 0.72-1.18 | 0.60 |
| **Blood glucose (1SD higher)** | **1.29** | **1.03-1.59** | **0.03** |
| **Diabetes duration (1 year longer)** | **1.03** | **1.01-1.06** | **0.0015** |
| **HbA1c <6.5%** | **2.97** | **1.33-7.25** | **0.007** |
| 6.5≦ HbA1c <7.0% | 1.77 | 0.73-4.54 | 0.21 |
| **7.0≦ HbA1c <7.5%** | **1.00** | **Reference** | |
| 7.5≦ HbA1c <8.5% | 1.62 | 0.70-4.03 | 0.26 |
| HbA1c ≧8.5% | 1.93 | 0.85-4.79 | 0.12 |

1: HR: hazard ratio, 2: CI: confidence interval, 3: SBP: systolic blood pressure, 4: SD: standard deviation, 5: LDL-C: low density lipoprotein-cholesterol, 6: HDL-C: high density lipoprotein-cholesterol

**Model 2**

|  | HR^1^ | 95%CI^2^ | P-value |
| --- | --- | --- | --- |
| Age (1 year older) | 1.02 | 0.99-1.05 | 0.27 |
| Male gender | 1.39 | 0.75-2.71 | 0.31 |
| Beta-blockers | 1.47 | 0.89-2.49 | 0.13 |
| **Ejection fraction (1SD^3^ higher)** | **0.61** | **0.50-0.77** | **<0.001** |
| Hemoglobin (1SD higher) | 0.74 | 0.55-1.00 | 0.051 |
| **Blood glucose (1SD higher)** | **1.32** | **1.02-1.67** | **0.034** |
| **eGFR^4^ (1SD higher)** | **0.63** | **0.47-0.85** | **0.002** |
| **Diabetes duration (1 year longer)** | **1.03** | **1.00-1.05** | **0.035** |
| Insulin use | 1.37 | 0.79-2.37 | 0.26 |
| **HbA1c <6.5%** | **2.85** | **1.22-7.16** | **0.015** |
| 6.5≦ HbA1c <7.0% | 1.13 | 0.43-3.03 | 0.81 |
| **7.0≦ HbA1c <7.5%** | 1.00 | **Reference** | |
| 7.5≦ HbA1c <8.5% | 1.24 | 0.51-3.17 | 0.64 |
| HbA1c ≧8.5% | 1.84 | 0.80-4.59 | 0.15 |

1: HR: hazard ratio, 2: CI: confidence interval, 3: SD: standard deviation, 4: eGFR: estimated glomerular filtration rate

**Additional Table S3: Univariate-unadjusted and multivariate-adjusted Cox proportional hazard analysis for predicting cardiovascular mortality (Continuous variables)**

|  | Univariate-unadjusted | | | Multivariate-adjusted | | |
| --- | --- | --- | --- | --- | --- | --- |
|  | HR^1^ | 95% CI^2^ | P-value | HR | 95% CI | P-value |
| Age, 1SD^3^ higher | 1.70 | 1.33-2.18 | **<0.001** | 1.13 | 0.84-1.52 | 0.41 |
| HbA1c, 1SD higher | 0.98 | 0.79-1.20 | 0.86 | 1.01 | 0.77-1.29 | 0.96 |
| Blood glucose, 1SD higher | 1.27 | 1.05-1.50 | **0.016** | 1.32 | 1.03-1.66 | **0.031** |
| Hemoglobin, 1SD higher | 0.51 | 0.41-0.64 | **<0.001** | 0.71 | 0.54-0.94 | **0.016** |
| Diabetes duration, 1SD higher | 1.60 | 1.30-1.94 | **<0.001** | 1.33 | 1.04-1.68 | **0.026** |
| eGFR^4^, 1SD higher | 0.49 | 0.38-0.62 | **<0.001** | 0.66 | 0.49-0.88 | **0.004** |
| Ejection fraction, 1SD higher | 0.57 | 0.47-0.70 | **<0.001** | 0.63 | 0.52-0.78 | **<0.001** |

1: HR: hazard ratio, 2: CI: confidence interval, 3: SD: standard deviation, 4: eGFR: estimated glomerular filtration rate
